# Interfacial Reaction and Electromigration Failure of Cu Pillar/Ni/Sn-Ag/Cu Microbumps under Bidirectional Current Stressing

**DOI:** 10.3390/ma16031134

**Published:** 2023-01-28

**Authors:** Zhiwei Fu, Jian Chen, Pengfei Zhao, Xiaotong Guo, Qingzhong Xiao, Xing Fu, Jian Wang, Chao Yang, Jile Xu, Jia-Yue Yang

**Affiliations:** 1 School of Energy and Power Engineering, Shandong University, Jinan 250100, China; 2 Science and Technology on Reliability Physics and Application of Electronic Component Laboratory, China Electronic Product Reliability and Environmental Testing Research Institute, Guangzhou 510610, China; 3 School of Microelectronics, Xi’an Jiaotong University, Xi’an 710049, China; 4 Jiaxing Key Laboratory of Flexible Electronics Based Intelligent Sensing and Advanced Manufacturing Technology, Institute of Flexible Electronics Technology of THU, Jiaxing 314000, China; 5 Optics & Thermal Radiation Research Center, Institute of Frontier and Interdisciplinary Science, Shandong University, Qingdao 266237, China

**Keywords:** bidirectional current, microbumps, electromigration, Cu-Sn compound

## Abstract

The electromigration behavior of microbumps is inevitably altered under bidirectional currents. Herein, based on a designed test system, the effect of current direction and time proportion of forward current is investigated on Cu Pillar/Ni/Sn-1.8 Ag/Cu microbumps. Under thermo-electric stressing, microbumps are found to be susceptible to complete alloying to Cu6Sn5 and Cu3Sn. As a Ni layer prevents the contact of the Cu pillar with the solder, Sn atoms mainly react with the Cu pad, and the growth of Cu_3_Sn is concentrated on the Cu pad sides. With direct current densities of 3.5 × 10^4^ A/cm^2^ at 125 °C, the dissolution of a Ni layer on the cathode leads to a direct contact reaction between the Cu pillar and the solder, and the consumption of the Cu pillar and the Cu pad shows an obvious polarity difference. However, with a bidirectional current, there is a canceling effect of an atomic electromigration flux. With current densities of 2.5 × 10^4^ A/cm^2^ at 125 °C, as the time proportion of the forward current approaches 50%, a polarity structural evolution will be hard to detect, and the influence of the chemical flux on Cu-Sn compounds will be more obvious. The mechanical properties of Cu/Sn3.0Ag0.5Cu/Cu are analyzed at 125 °C with direct and bidirectional currents of 1.0 × 10^4^ A/cm^2^. Compared with high-temperature stressing, the coupled direct currents significantly reduced the mechanical strength of the interconnects, and the Cu-Sn compound layers on the cathode became the vulnerable spot. While under bidirectional currents, as the canceling effect of the electromigration flux intensifies, the interconnect shear strength gradually increases, and the fracture location is no longer concentrated on the cathode sides.

## 1. Introduction

The interconnection of Cu pillar microbumps has excellent conductivity and a smaller pitch. It has been widely used in advanced packaging products such as flip-chip, Co-Wos, and microsystems [1,2]. At present, the interconnection height of Cu pillar microbumps does not exceed 100 μm, and the pitch can be reduced to less than 50 μm, significantly improving the chip package I/O density [3,4]. However, the current density applied to microbumps increases dramatically with the reduction of interconnect size, making electromigration failure of interconnects significant. In addition, the solder in microbumps is prone to alloying under heavy current densities, which in turn forms micro-voids and cracks at weld interfaces, seriously reducing the mechanical properties of interconnection [5,6,7,8]. Research on Cu pillar microbumps has mainly focused on electromigration under constant direct current (DC) without considering the influence of transformation in the current direction. Ma et al. investigated the electromigration behavior of Cu pillar microbumps and found that pure Sn solder was susceptible to complete alloying into Cu_6_Sn_5_ and Cu_3_Sn layers [9]. Park et al. investigated the electromigration performance of Cu pillar/Ni/Sn-Ag to analyze the potential mechanisms by which Ni layers improve the resistance to electromigration. Xu and Fu et al. investigated the effect of grain orientation on electromigration properties and found that grain orientation rotated in response to electron flow [10,11,12]. In previous work [13,14,15], experimental and theoretical studies on Cu pillar microbumps were carried out and determined structural evolution, failure mechanisms, and life prediction models of lead-free solder under the micro-size effect. We found that the dissolution of the cathodic Ni layer is a critical failure mode for Cu Pillar/Ni/Sn-Ag/Cu micro-interconnect. In addition, rapid alloying of Sn solder and polarity depletion of Cu pads always exist. However, all the work was carried out under DC stress, ignoring the influence of current direction and the time proportion of forward current on the above phenomena. Solder joints may carry unidirectional or bidirectional current (BC) stress during their service [16,17,18,19]. Therefore, it is inadequate to use only direct current to evaluate the electromigration reliability. Many studies reveal that relevant failure mechanisms and reliability under alternating or pulse direct current (AC or PDC) show significant differences. C. Basaran et al. simulated thermo-electric fields in the SAC405 microbump and pointed out that although it still exhibits an electromigration failure mechanism, the solder joint would have a longer electromigration life under the low-frequency pulsed current [20]. Yao et al. revealed that skin effects would occur on microbumps under a high-frequency current, and the surface of the solder joint showed a more obvious electromigration phenomenon [21]. Zhu et al. found that a low-frequency AC current also reduced mechanical strength and caused the “temperature cycling” thermal effect on microbumps [22,23].

However, there is still a vacuum in our knowledge regarding the electromigration behavior of Cu pillar microbumps under bidirectional current stressing, and the precise failure mechanism is unclear and requires further investigation. In this work, a DC/PDC electromigration test system has been exploited that is compatible with direct and bidirectional pulsed current loading. Cu Pillar/Ni/Sn-Ag/Cu micro-interconnect test chips with a daisy-chain structure are designed and fabricated. Electromigration contrast experiments were conducted to investigate the effects of the current direction and time proportion of a forward current on the structural evolution of Cu pillar microbumps under direct and bidirectional currents. A mechanical test structure for solder joint interconnection was constructed to analyze the differences in shear strength under high temperature, direct, and bidirectional pulsed current stressing. The relevant experimental data and conclusions are essential for evaluating the reliability of Cu pillar microbumps under actual service conditions.

## 2. Test System Development and Sample Production

### 2.1. Development of the DC/PDC Electromigration Test System

**Figure 1:**
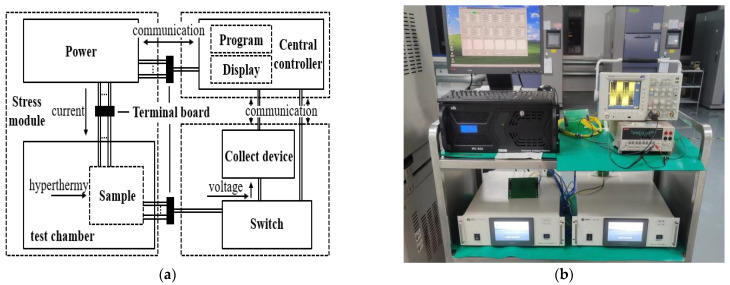
DC/PDC electromigration test system. (**a**) Schematic diagram of test system; (**b**) Electromigration test system.

The DC/PDC electromigration test system has been developed based on the multifunctional adjustable power supply and is compatible with direct and bidirectional pulsed current loading. As shown in Figure 1, the test system is mainly divided into three modules, including stress loading, central control, and data acquisition. The central control module is connected to the drive power supply using an RS-485 port to communicate synchronously with the data acquisition and switching arrays. The samples in the temperature test chamber are connected to the drive power supply in Kelvin. Parameters can be set in the control module to regulate the output current of the power supply and switching arrays can dynamically switch the data acquisition channels. The voltage waveform of samples is collected in real-time using an oscilloscope, and relevant algorithms are designed to analyze the waveform information for obtaining real-time resistance.

### 2.2. Fabrication of Micro-Solder Joint Interconnection Samples

**Figure 2:**
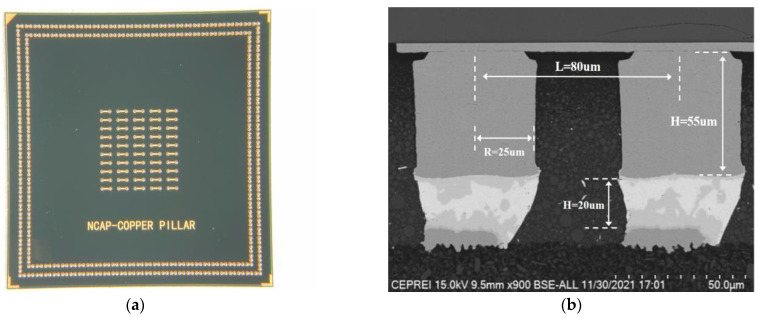
Cu pillar microbumps with a daisy chain structure. (**a**) Micrograph of a bare chip; (**b**) SEM image of Cu pillar microbumps.

Cu Pillar/Ni/Sn-1.8 Ag/Cu micro-interconnect test chips with a daisy-chain structure were designed and fabricated. Optical microscopy and scanning electron microscopy (SEM) images of the test sample are shown in Figure 2. The radius and height of the Cu pillar are 25 μm and 55 μm, respectively. The Ni layer has a thickness of approximately 2 μm, the size of the solder is approximately 20 μm, and the interconnection pitch of Cu bumps is 80 μm. The bare chips were first welded to the substrate, and then filling and plastic sealing operations were conducted to form the test chips. After the test, epoxy resin was used to seal the daisy chain structure samples, and after sandpaper grinding, Al powder polishing, and etching, the scanning electron microscope was used to observe the micromorphology.

**Figure 3:**
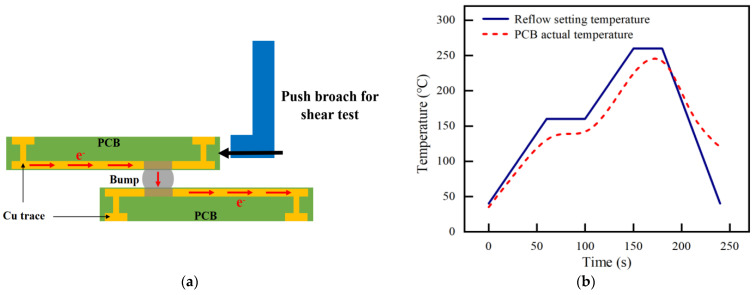
(**a**) Structure of the Cu/Sn3.0Ag0.5Cu/Cu sample. (**b**) Reflow welding temperature curves.

The samples shown in Figure 2 are not suitable for mechanical properties testing. Therefore, the mechanical testing structure for solder joint interconnection was constructed to analyze the differences in shear strength under high temperature and a direct and bidirectional pulsed current. As shown in Figure 3a, the samples consist of a Cu/Sn3.0 Ag0.5Cu/Cu interconnect structure composed of two PCB boards overlapped with Sn3.0Ag0.5Cu solder balls with a diameter of approximately 100 μm. The testing structures were first preheated after 60 s of heating to 160 °C during the welding process, then heated to 260 °C for reflow soldering, and finally cooled down. Specific reflow soldering process curves are shown in Figure 3b, which shows the welding temperature curves set in the system and the actual temperature change curves of the PCB board, respectively. During the actual welding process, the temperature rise of the samples will be lower than the set values, and the temperature change process will also lag behind the set values.

## 3. Results and Discussion

### 3.1. Differences in Microstructural Evolution under Direct and Bidirectional Currents

**Figure 4:**
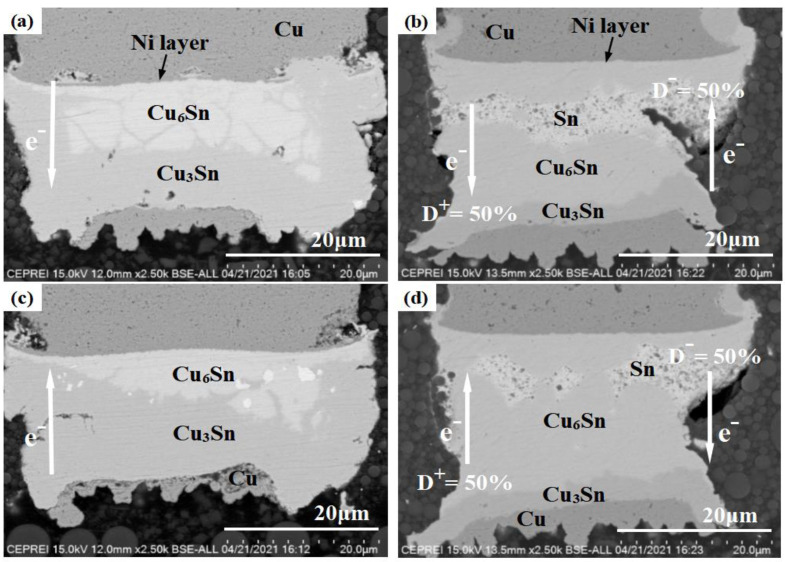
SEM images of Cu pillar microbumps at 125 °C, 3.5 × 10^4^ A/cm^2^. (**a**,**c**) DC stress; (**b**,**d**) BC-50% stress.

Figure 4 shows the SEM images of adjacent Cu pillar microbumps at 125 °C and an applied current density of 3.5 × 10^4^ A/cm^2^. Both direct and bidirectional stress currents are loaded for 220 h, and the frequency of the pulse is 1 Hz. Here, the anode and cathode are defined when the current direction is positive. Figure 4 shows a set of SEM images taken under the same experimental conditions, such as (a,c) and (b,d), are respectively, solder joints on both sides of the adjacent Cu pillar microbumps shown in Figure 2. The magnitudes of the forward and backward currents are identical, and the time proportion of the forward current is 50% (D^+^ = D^−^ = 50%).

As shown in Figure 4a,c, the lead-free solder has been alloyed completely to form Cu_6_Sn_5_ and Cu_3_Sn layers under DC stress. The cathodic Ni layer in Figure 4a shows localized dissolution and depletion in several places. In contrast, the anodic Ni layer in Figure 4c still maintains its complete layered morphology, isolating the Cu pillar from direct contact with the solder. As electrons migrate from the cathodic Ni layer to the anodic Cu pad sides in Figure 4a, the electron wind will drive Ni to dissolve continuously into Sn-1.8 Ag solder [24]. Without the protection of the Ni layer, the Cu pillar will react with the solder. This process consumes plenty of Sn atoms, effectively slowing down the consumption of Cu pads on the anode sides. The degree of anodic Cu pad depletion is thus significantly greater in Figure 4c than that in Figure 4a.

In Figure 4b,d, the alloying process of microbumps under a bidirectional current stress had obviously slowed down. The Sn-1.8 Ag solder remains under the same test time, and the consumption of the Cu pad and the growth of the Cu_3_Sn layer are also significantly less than under the DC stress condition. Moreover, both the cathodic and anodic Ni layers still maintain complete layered morphology, and the adjacent interconnected microbumps show similar structural evolution patterns. When electrons flow from the Ni layer to the Cu pad, the electron wind forces the Ni layer to migrate into the solder in the same direction as the chemical diffusion, which causes the Ni layer dissolution process to be accelerated. On the other hand, the Cu-Sn compound near the Ni layer will be dissolved by the electron wind, and Cu atoms will migrate to the Cu pad sides, promoting the growth of the anodic Cu-Sn compound layers. However, when electrons flow from the Cu pad to the Ni layer, the electromigration of Ni atoms is in the opposite direction from the chemical diffusion, inhibiting the dissolution process of the Ni layer. The Cu-Sn compound near the Cu pad dissolves under electron wind forces, and Cu atoms migrate towards the Ni layer, inhibiting the growth of Cu-Sn compound layers on the Cu pad sides. Under bidirectional current stress, the dissolution of the Ni layer and the anodic Cu-Sn compound growth process are repeatedly accelerated and inhibited. When the time proportion of the forward current reaches a specific value, the acceleration of solder alloying by the electromigration effect will be substantially counteracted. Thus, converting the Cu pillar microbumps to Cu-Sn compounds will take a longer time, and the microstructure evolution will not show significant polar differences. Notably, the Ni layer can provide adequate protection for the Cu pillar, as the Ni-Sn reaction requires higher activation energy than the Cu-Sn reaction [25]. Therefore, through the above analysis, it can be concluded that properly increasing the thickness of the Ni layer will effectively prolong the corrosion time of the Cu pillar and improve the service life of the interconnection. Whether direct or bidirectional currents, Sn atoms mainly react with the Cu atoms of the Cu pad, and the growth of Cu_3_Sn layers is concentrated on the Cu pad sides.

### 3.2. Influence of the Forward Current’s Time Proportion on Structural Evolution

**Figure 5:**
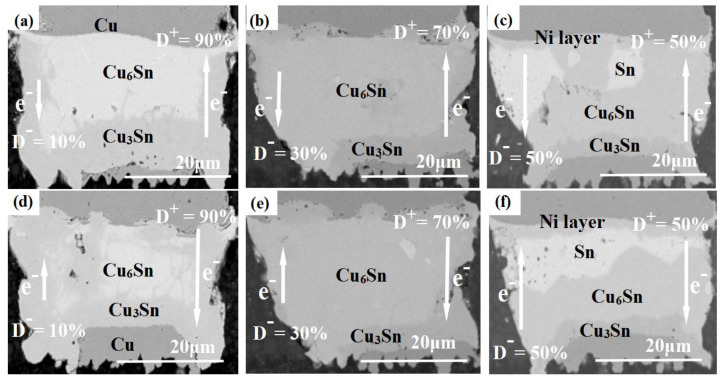
SEM images of Cu pillar microbumps at 125 °C, 2.5 × 10^4^ A/cm^2^. (**a**,**d**) D^+^ = 90%; (**b**,**e**) D^+^ = 70%; and (**c**,**f**) D^+^ = 50%.

Figure 5 shows the SEM images of adjacent interconnected microbumps at 125 °C and an applied current density of 2.5 × 10^4^ A/cm^2^. All the current stress is bidirectional and has a pulse frequency of 100 Hz. Figure 5 shows a series of SEM images taken under the same experimental conditions, such as (a,d), (b,e), and (c,f) which are solder joints on both sides of the adjacent Cu pillar microbumps shown in Figure 2. The time proportion of the forward current in Figure 5a,d, is 90% (D^+^ = 90%), and those in Figure 5b,c, and Figure 5e,f, are 70 and 50%, respectively.

As shown in Figure 5a,d, when D^+^ = 90%, the structural evolution of Cu pillar microbumps is still obviously influenced by the electromigration effect, and the consumption of Ni layers and Cu pads shows polar differences. In contrast, the Ni layers and Cu pads in adjacent Cu pillar microbumps in Figure 5b,c,e,f are relatively close in depletion, with no significant polar differences in structural evolution. When the time proportion of the forward current reduces to 70%, the layered morphology of the cathodic and anodic Ni layers in Figure 5b,e are destroyed, and localized corrosion of the Cu pillar occurs. When D^+^ = 50%, the Ni layer in Figure 5c,f maintains its complete layered morphology, effectively protecting the Cu pillar. When D^+^ = 90% and D^+^ = 70%, the solder has been completely alloyed into Cu_6_Sn_5_ and Cu_3_Sn. However, when D^+^ = 50%, there is still plenty of Sn remaining in the solder. The acceleration effect of current stress on the solder alloying process is greatly reduced. It is predictable that with the extension of the test time, when D^+^ = 50%, the Ni layer will also be consumed without polarity difference.

Under temperature stress, the chemical diffusion of Cu and Ni atoms in the Cu pillar microbumps occurs due to a concentration gradient difference, and the chemical diffusion flux can be expressed by the following equation [26]:

(1) $$J_{chem}=-D\frac{dC}{dx}$$

where *D* is the diffusivity, *C* is the atomic concentration, and *x* is the diffusion distance, respectively, with the negative sign indicating that diffusive motion always follows the direction of the concentration gradient. The electromigration flux of Cu and Ni atoms due to the electron wind under current stress can be expressed as follows [27]:

(2) $$J_{em}=C\frac{D}{kT}Z^{*}e\rho j$$

where Z* is the effective charge number, *e* is the electron charge, *ρ* is the resistivity of solder, *j* is the current density, *k* is the Boltzmann constant, and *T* is the temperature in the unit of Kelvin, respectively. Thus, while coupling with direct current, the atomic flux can be expressed by the following equation:

(3) $$J=-D\frac{dC}{dx}+C\frac{D}{kT}Z^{*}e\rho \left|2D^{+}-1\right|•j.$$

It can be concluded from Equation (3) that the atomic movement under thermoelectric stress is a composite effect of chemical diffusion. Equation (3) shows that the electromigration flux will be discounted under bidirectional currents, which can be equivalent to the current density being reduced to the original |2D^+^ − 1| times. When D^+^ = 70%, the bidirectional current density of 2.5 × 10^4^ A/cm^2^ can be equated to be a direct current density of 1.0 × 10^4^ A/cm^2^. The influence of the current on the evolution of the structure is significantly reduced. Therefore, if the time proportion of forward current is near 50% (|2D^+^ − 1| is closer to zero), the polarity evolution of the Ni layer and the Cu pad is harder to observe, which will greatly improve the interconnection life.

### 3.3. Differences in Mechanical Properties under Temperature and Current Stress

Based on the Cu/Sn3.0Ag0.5Cu/Cu interconnect structure overlapped in Figure 3, experiments were conducted at 125 °C with and without a coupling current density of 1.0 × 10^4^ A/cm^2^ for 100 h. The current stress loading includes direct current (DC) and a bidirectional pulsed current of D^+^ = 90% (BC-90%) and D^+^ = 50% (BC-50%). Seven samples were tested under each set of conditions, and the shear strength of solder joints was analyzed.

**Figure 6:**
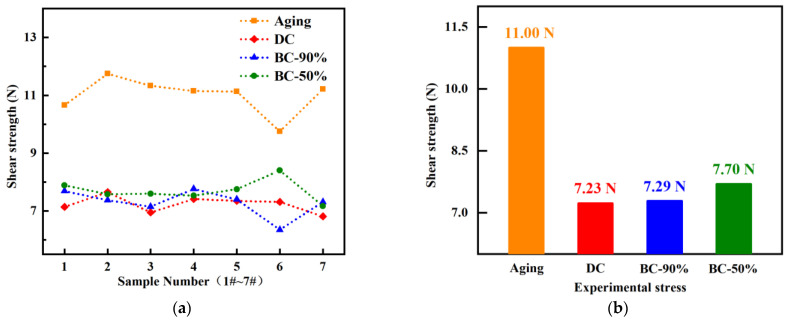
Shear strength of solder joint interconnections under different experimental stresses. (**a**) Shear strength statistics. (**b**) Average shear strength.

Figure 6a shows the shear strength’s numerical distribution at the solder joints under different stresses, and it can be observed that the Cu/Sn3.0Ag0.5Cu/Cu interconnect samples have good consistency in the welding process. Figure 6b shows the average shear strength from high to low in order is Aging > BC-50% > BC-90% > DC. The average shear strength after 100 h at 125 °C is 11.0 N. After a coupling DC of 1.0 × 10^4^ A/cm^2^, the shear strength is reduced to 7.23 N, with a drop of up to 34.3%. The current significantly degrades the mechanical properties of the solder joints. On the other hand, the switching of the current direction slows down the degradation of mechanical properties. When D^+^ = 90% and D^+^ = 50%, the average shear strengths are 7.29 N and 7.70 N, respectively. It can be summarized that the shear strength of solder joints increases slightly as |2D^+^ − 1| approaches zero. It is also worth noting that although the increase in mechanical properties of solder joints under experimental conditions is subtle, the actual device often uses a large number of solder joints in an array of interconnections, so the holistic change in mechanical properties cannot be ignored.

**Figure 7:**
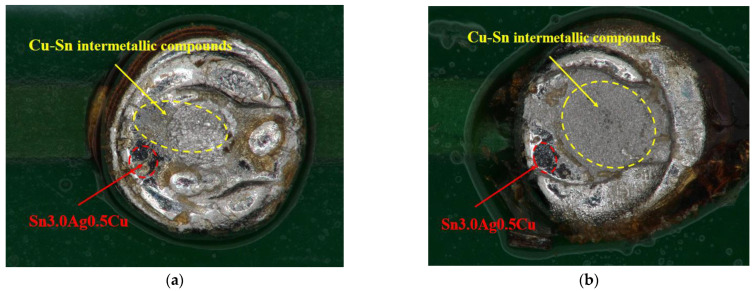
Fracture surfaces of Cu/Sn3.0Ag0.5Cu/Cu. (**a**) The cathode side. (**b**) The anode side.

A group of broken solder joint interfaces after a BC-90% test was photographed and analyzed. The fracture surfaces of Cu/Sn3.0Ag0.5Cu/Cu are shown in Figure 7. In the same way, the anode and cathode are defined when the current direction is positive. It is observed that the fracture of both the cathode and the anode occurs mainly on the Cu-Sn compound layers. The Cu-Sn compound has a brittle structure and is an easily vulnerable spot for interconnections. Moreover, there are significantly more Cu-Sn compounds at the fracture position of the anode than at the cathode. This is mainly because the current accelerates the growth of the Cu-Sn compounds on the anode sides.

The fracture location distribution of all the samples after the test is shown in Table 1. As Table 1 shows, under high-temperature and BC-50% conditions, the fracture locations are almost uniformly distributed at both ends of the solder joints. While coupling DC and BC-90% conditions, the fracture location of the solder joint is mainly concentrated on the cathode layers. The differences in shear strength and fracture location in Figure 6 and Table 1 are mainly related to the growth of the Cu-Sn compound layers under different stresses. Under current stress, metal atoms in the solder joints will migrate directionally from the cathode to the anode side and will form microvoids at the cathode interface. As the stress time increases, the cathode voids gradually expand into cracks, reducing the mechanical strength of the interconnected structure. Therefore, the weakness under DC and BC-90% conditions is mainly concentrated on the cathodic Cu-Sn compound layers. In contrast, under high temperature and BC-50% conditions, the Cu-Sn compound layers did not show a significant difference in polar growth because of a lack of current acceleration or an existing canceling effect of electromigration flux. The fracture locations of solder joints appeared randomly at the ends of the Cu-Sn compound layers.

## 4. Conclusions

In this work, interfacial reaction and electromigration failure of Cu pillar microbumps are investigated under direct and bidirectional currents. The main conclusions are as follows:

(1) The Sn atoms mainly react with the Cu pad in Cu Pillar/Ni/Sn-Ag/Cu interconnections, and the growth of the Cu_3_Sn layers is concentrated on the Cu pad sides. While under bidirectional pulsed currents,, the electromigration flux is only |2D^+^-1| times that of DC, and the complete conversion time of microbumps into the Cu-Sn compound is prolonged. When under a pulsed current of 2.5 × 10^4^ A/cm^2^ at 125 °C (D^+^ = 70%), the structural evolution no longer shows significant polarity differences;
(2) The mechanical strength of a Cu/Sn3.0Ag0.5Cu/Cu interconnection is ranked as aging > BC-50% > BC-90% > DC, and the Cu-Sn compound layer is the mechanically weak link. Direct currents will significantly reduce the mechanical strength of the interconnection. While under high temperatures and bidirectional currents, the degradation of mechanical strength is alleviated because of the lack of current acceleration or an existing canceling effect of electromigration flux. Shear strength increases slightly as |2D^+^-1| approaches zero, and the fracture location is no longer concentrated on the cathodic Cu-Sn compound.

## Figures and Tables

**Table 1:** Distribution of shear fracture locations.

	Stress	Aging	DC	BC-50%	BC-90%
Fracture Location					
Anode		3	0	3	1
Cathode		4	7	3	6
Middle position		0	0	1	0

## Data Availability

The data will be available on reasonable request.
